# Urgent Need for Pairing Training and Education With Fentanyl Test Strip Distribution

**DOI:** 10.1111/dar.70049

**Published:** 2025-10-13

**Authors:** Megan K. Reed, Tracy Esteves Camacho, Kristin L. Rising, Rose Laurano, Danielle Albaciete, Stephen E. Lankenau

**Affiliations:** ^1^ Department of Emergency Medicine, Sidney Kimmel Medical College Thomas Jefferson University Philadelphia Pennsylvania USA; ^2^ Center for Connected Care, Sidney Kimmel Medical College Thomas Jefferson University Philadelphia Pennsylvania USA; ^3^ College of Population Health Thomas Jefferson University Philadelphia Pennsylvania USA; ^4^ Hackensack Meridian School of Medicine Nutley New Jersey USA; ^5^ Department of Community Health and Prevention Drexel University Philadelphia Pennsylvania USA

**Keywords:** drug testing, FTS, harm reduction, immunoassay

## Abstract

**Introduction:**

Fentanyl test strips (FTS) are increasingly recommended for non‐heroin drugs to detect potential fentanyl adulteration. The aim of this study was to better understand how people are using FTS.

**Methods:**

In simulation exercises between September and October 2023, 40 people who used drugs participated in a mock use of FTS on a simulated drug, interpreted FTS results and completed interviews about their FTS use. Data were analysed in NVivo.

**Results:**

Prior to study enrolment, 80% of participants reported receiving training on using FTS, some had instructed others on their use, and 71% reported a positive result at the last use of a FTS on a non‐heroin drug. During the simulation exercise, none of the participants used FTS as recommended: most under‐diluted the sample or used the FTS in another manner not indicated. During the interpretation of FTS strip results, 45% correctly interpreted a positive test; 55% a negative test with clear lines; and 30% a negative test with a faint second line.

**Discussion and Conclusions:**

Observed errors in FTS simulations would likely lead to false positive results. Findings highlight the lack of appropriate training people have received and the need to develop educational approaches to ensure people use FTS properly to optimise their impact.

## 1 Introduction

In 2022, 107,941 drug overdose deaths occurred in the United States, with nearly 88% of deaths attributable to synthetic opioids, including fentanyl [1]. Deaths involving both opioids and stimulants represent an increasing portion of overdose deaths, and it is hypothesised that most of these deaths are due to intentional co‐ingestion of these substances. However, case reports indicate that some adulteration of the non‐heroin/non‐fentanyl drugs is occurring, resulting in unintentional consumption of opioids and subsequent overdoses [2, 3, 4, 5]. When this occurs, it is unclear whether the combination of fentanyl with another drug is attributable to systematic intentional adulteration, widespread unintentional cross‐contamination at the point of packaging, or isolated incidents. In Philadelphia, a major hub of the overdose crisis, there were 1413 overdose deaths in 2022, and fentanyl was the most involved substance. Of all overdoses, more than half (55%) involved both an opioid and a stimulant [6].

Fentanyl test strips (FTS) are one harm reduction tool that can mitigate the harm associated with using drugs adulterated with fentanyl. Originally developed for urine drug screening, FTS have been repurposed to inform people who use drugs (PWUD) of the presence of fentanyl in the drugs they consume [7]. Prior studies have found that many PWUD who use FTS prior to consuming drugs change their drug use behaviour, such as reducing the amount of drugs used if it tests positive for fentanyl, advising social networks on unwanted FTS results, and changing the mode of administration [8, 9, 10]. Given the ubiquity of fentanyl in the heroin/fentanyl supply and given the uncertainty of how and when fentanyl adulteration of non‐heroin/fentanyl drugs is occurring, FTS use is increasingly recommended for non‐heroin/fentanyl drugs.

While the literature on FTS has grown in recent years, there is little understanding to date about the actual procedures that PWUD use to test drugs with FTS or the accuracy with which PWUD interpret results. This information about FTS use can inform future training to optimise the impact of FTS as a harm reduction tool. To that end, the purpose of this study was to gain understanding about how PWUD are using FTS on non‐heroin/fentanyl drugs and how they interpret results.

## 2 Methods

Between September and October 2023, we conducted 40 interviews with PWUD who reported use of non‐heroin/fentanyl substances. Participants were recruited through street intercept at busy intersections in the Kensington neighbourhood of Philadelphia, an area widely known as an open‐air drug market. To be eligible, participants needed to be 18 years or older and have a history of drug use including substances other than heroin/fentanyl. To capture a range of perspectives, we recruited both people who had previous experience using FTS (*n* = 31) and those who had never used FTS (*n* = 9). Participants provided verbal consent before participating. The interview process consisted of three stages: (i) a pre‐simulation interview discussing history and current FTS use; (ii) a simulation where they demonstrated FTS use on simulated drugs; and (iii) a post‐simulation interview reflecting on the simulation experience. Interviews were conducted in a community space close to the area of recruitment, with only participants and up to two study team members present. Participants received $50 cash as study .remuneration. This study was approved by the Thomas Jefferson University Institutional Review Board.

**FIGURE 1:**
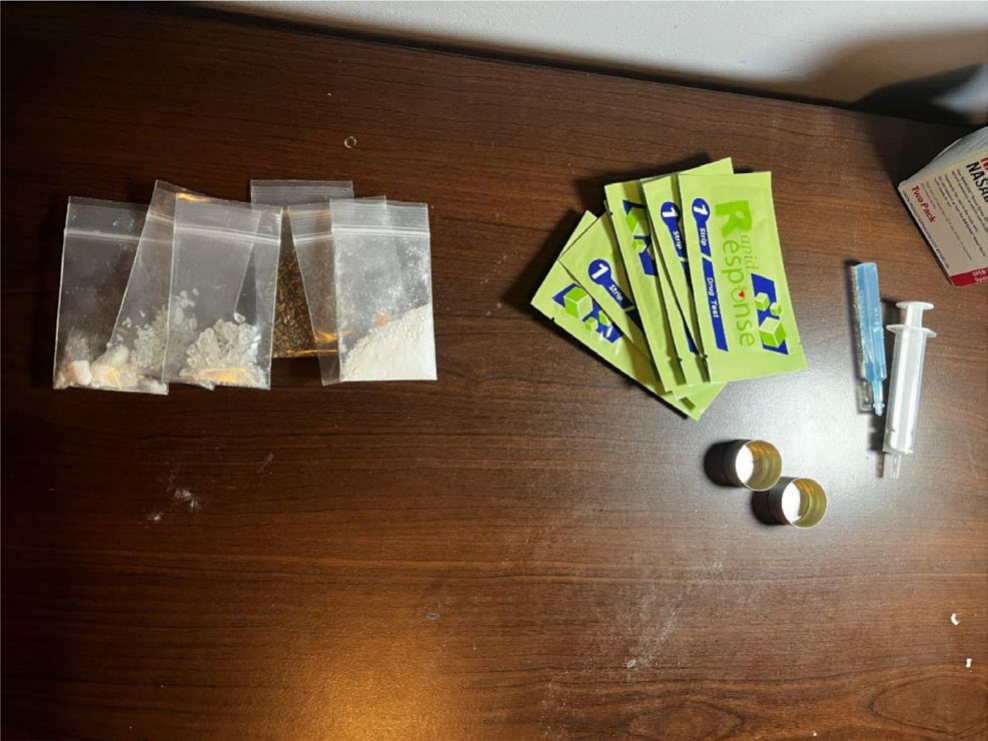
Simulation drugs used for fentanyl test strip simulation.

**FIGURE 2:**
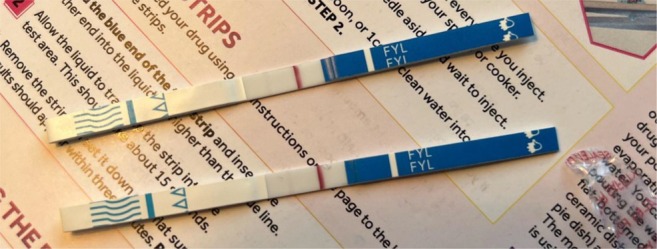
Image of fentanyl test strip results—Strip 1, faint negative.

**FIGURE 3:**
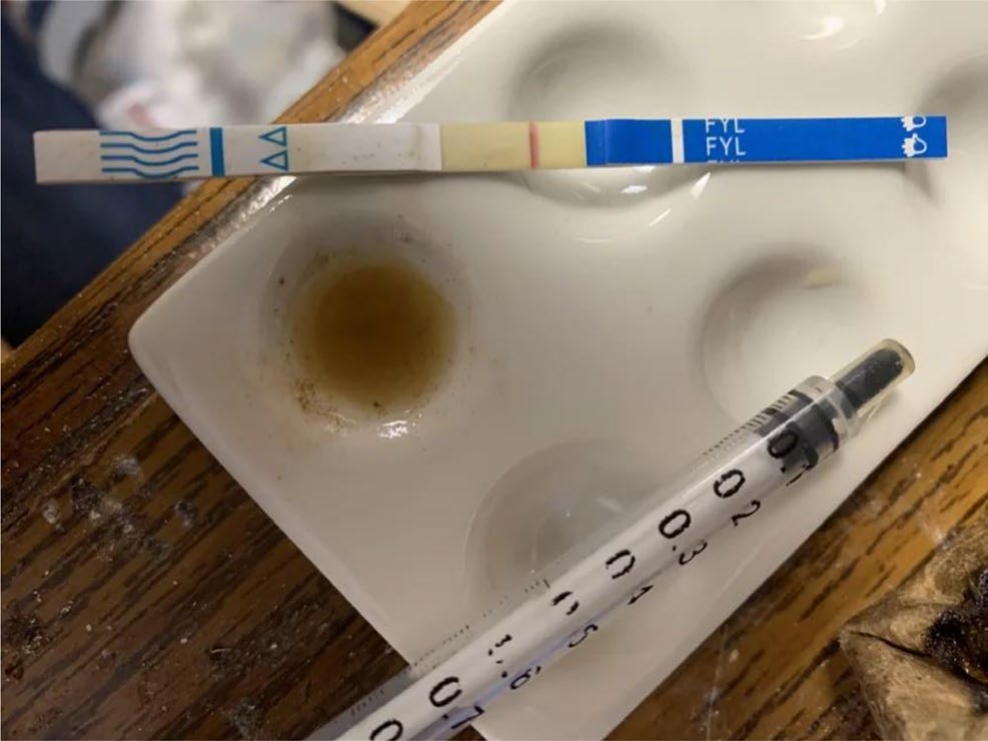
Image of fentanyl test strip results—Strip 2, positive.

**FIGURE 4:**
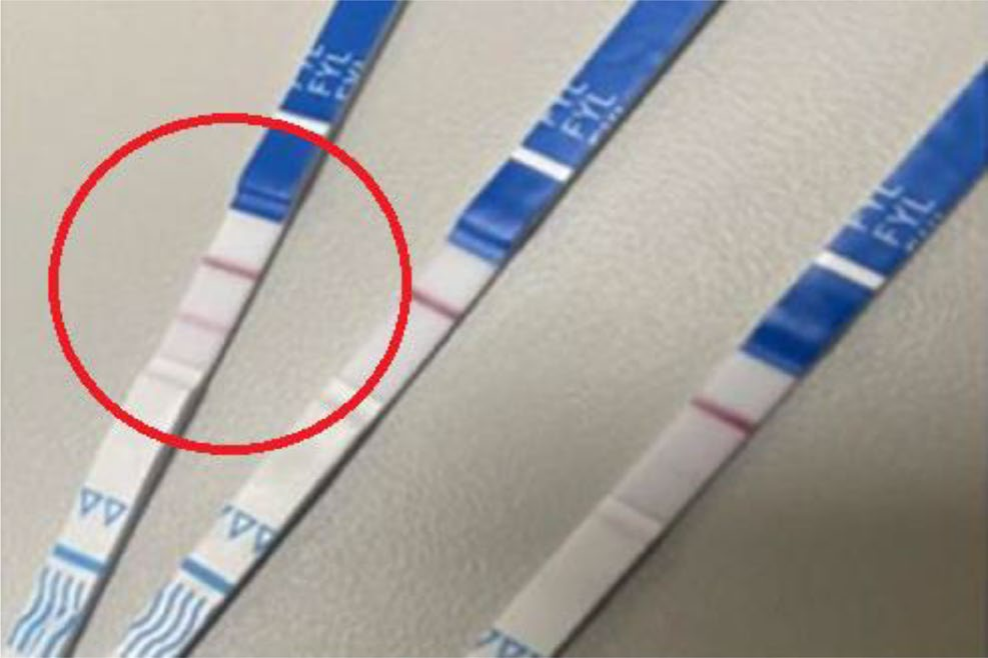
Image of fentanyl test strip results—Strip 3, negative.

Interviews were led by authors MKR, TEC and DA. There were two pre‐simulation interview guides tailored to participants with and without prior FTS experience, covering topics such as their knowledge and experience with FTS, decision‐making related to FTS, the environment of FTS use, interactions with law enforcement and experience using xylazine test strips. During the simulation, participants selected one of three settings that most closely resembled their usual drug testing environment: a kitchen table (private residence), a tent (semi‐sheltered area) or the floor of an empty room (public sidewalk). Participants were provided with sealed FTS, commonly used supplies for testing (water, cookers, syringes) and a simulated drug representing their preferred substance to test for fentanyl (see Figure 1). Simulated drugs offered were powder cocaine (powdered sugar), crack cocaine (rock salt), methamphetamine (crushed rock candy), prescription pills (over‐the‐counter pill) and synthetic cannabinoids (herbal smoking blend). During the simulation, participants could use any supplies provided by the study or anything else (e.g., supplies obtained from a syringe exchange previously, bottle caps from water in their bags) they had with them. Participants used the FTS and vocalised their thoughts and decision‐making process during the simulation, while the interviewer made notes about their process on a form that included information on the dilution of the drug, how they used the FTS and how they interpreted the results. The correct action for each of these was modeled after DanceSafe's instructions as of September 2023 (www.dancesafe.org). After the simulation, participants were interviewed to clarify their thought process. Additionally, participants were shown images of three FTS results and asked to interpret the results (see Figures 2, 3 and 4). After the interviews, participants were shown an instructional 5:14 video on proper FTS use.

The study team developed a codebook and 25% (*n* = 10) of the interviews were coded asynchronously by all coders TEC, DA, RL. Deductive codes derived from research questions and interview guides, and inductive codes emerging from the text were developed and utilised in the analysis of interviews through both directed and conventional content analysis methods [11, 12]. Weekly meetings were held to compare coding schemes. The remaining interviews were coded independently. A final score showing agreement between coders was a Cohen's kappa of 0.97, with a kappa of > 0.80 considered excellent. After coding all interviews, study team members reviewed each code's output and wrote summaries containing hyperlinks to the source text. The team met to discuss summaries and themes.

When reporting quotes, participants are identified by their selected pseudonym along with their race, gender and the drug they selected for the FTS simulation.

## 3 Results

Forty people with a history of drug use, including substances other than fentanyl/heroin, participated in an interview (Table 1). Sixty percent of the sample identified as White and 28% identified as Black. A quarter of the sample identified as Latino/Hispanic ethnicity. Half of the sample was male and the average age was 39.2 years (range 27 to 55 years old). Most people (98%) reported using heroin/fentanyl, xylazine/tranq (75%), crack cocaine (70%) and/or powder cocaine (65%) in the past month. Eighty‐five percent of participants had previously experienced an opioid overdose in their lifetime; 23% had experienced an overdose while using non‐opioid substances. Most participants reported being unhoused (63%). When asked to choose a simulation environment that mirrored where they would use FTS, 58% chose the street and the remainder chose indoors in an apartment or home. No participants opted to simulate testing in the tent. The imitation drugs selected for the FTS simulation included powder cocaine (35%), crack cocaine (25%), methamphetamine (23%), prescription pill (13%) and synthetic cannabinoids (“K2”/”spice”) (5%).

**TABLE 1:** Characteristics of participants simulating fentanyl test strips (FTS) use in Philadelphia, PA (*n* = 40).

	*n* (%)
Age, mean (SD)	39.2 (7.6)
Gender identity	
Male	20 (50.0)
Female	19 (47.5)
Transgender	1 (2.5)
Ethnicity	
Latino/Hispanic	10 (25.0)
Race	
White	24 (60.0)
Black	11 (27.5)
Native American or Alaska Native	2 (5.0)
Other^a^	6 (15.0)
Drugs used in past month	
Heroin/fentanyl	39 (97.5)
Xylazine/tranq	30 (75.0)
Crack cocaine	28 (70.0)
Powder cocaine	26 (65.0)
Methamphetamine	19 (47.5)
Benzodiazepine, not prescribed	9 (22.5)
Prescription opioids, not prescribed	7 (17.5)
Synthetic cannabinoids (K2/spice)	7 (17.5)
PCP/wet	5 (12.5)
Buprenorphine, not prescribed	3 (7.5)
Methadone, not prescribed	2 (5.0)
Other^b^	10 (25.0)
Drugs ever tested with FTS	
Heroin/fentanyl	30 (75.0)
Xylazine/tranq	13 (32.5)
Powder cocaine	11 (27.5)
Crack cocaine	11 (27.5)
Methamphetamine	11 (27.5)
Benzodiazepine, not prescribed	5 (12.5)
Prescription opioids, not prescribed	4 (10.0)
Other	2 (5.0)
None^c^	9 (22.5)
Overdose history	
While using opioid	34 (85.0)
While using non‐opioid	9 (22.5)
On any drug ever	35 (87.5)
Housing status	
Unhoused	25 (62.5)
Housed	11 (27.5)
Unstably housed^d^	4 (10.0)
Simulation environment	
Street	23 (57.5)
Apartment/home	17 (42.5)
Simulation drug	
Powder cocaine	14 (35.0)
Crack cocaine	10 (25.0)
Methamphetamine	9 (22.5)
Prescription pill	5 (12.5)
K2/Spice	2 (5.0)

^a^ All participants who selected their race as ‘Other’ identified as Latino/Hispanic.

^b^ Other drugs used in the last 30 days included cannabis, ketamine and gabapentin, not prescribed.

^c^ Other drugs tested with FTS included MDMA and cannabis.

^d^ Includes ‘couchsurfing’, or staying in temporary accommodations with friends or family.

### 3.1 History of Fentanyl Test Strip Use

**FIGURE 5:**
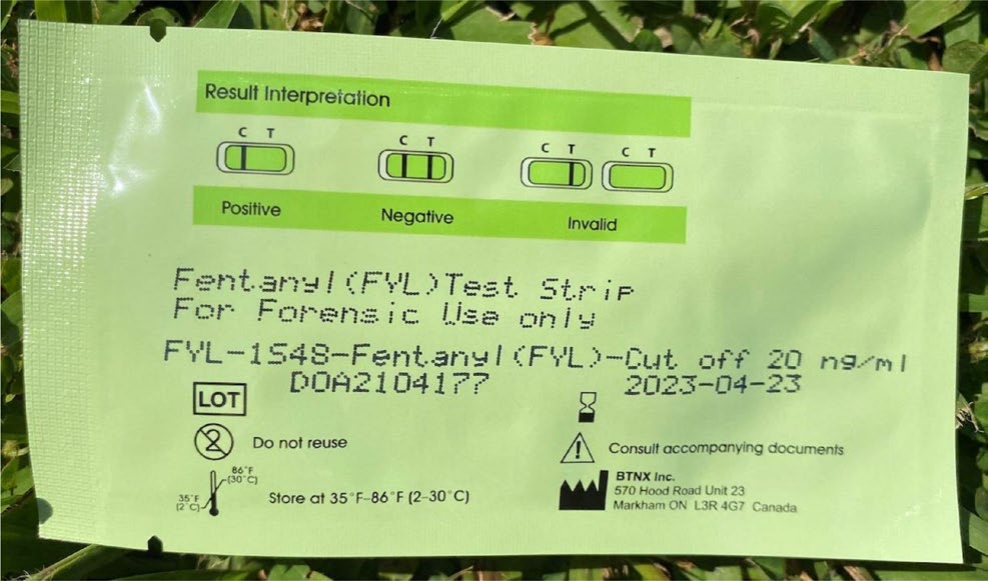
Example of BTNX fentanyl immunoassay packaging instructions.

When asked about where they first obtained FTS (Table 2), most participants (88%) reported getting FTS from a nonprofit organisation or street outreach, and only 30% reported receiving training from a nonprofit organisation or street outreach. Over a quarter of participants relied on the directions on the packaging or paper inserts for learning how to use FTS (Figure 5). Twenty percent of participants reported never receiving any FTS training.

**TABLE 2:** History of fentanyl test strip (FTS) use of participants simulating FTS use in Philadelphia, PA (*n* = 40).

	*n* (%)
First obtained FTS	
Nonprofit/street outreach	35 (87.5)
Social network	3 (7.5)
NA‐ never offered FTS	2 (5.0)
First FTS training	
Nonprofit/street outreach	12 (30.0)
FTS packaging/paper inserts	11 (27.5)
Social network	8 (20.0)
Online	1 (2.5)
NA‐ Never trained	8 (20.0)
Average years using FTS (*n* = 31), mean (SD)	1.7 (1.5)
Most recent non‐heroin/fentanyl drug tested with FTS (*n* = 31)	
Powder cocaine	9 (29.0)
Crack cocaine	6 (19.4)
Methamphetamine	4 (12.9)
Xylazine/tranq	1 (3.2)
Prescription opioids, not prescribed	1 (3.2)
Benzodiazepine, not prescribed	1 (3.2)
NA‐ only used on heroin	9 (29.0)
Most recent non‐heroin/fentanyl FTS result (*n* = 22)	
Positive	16 (72.7)
Negative	5 (22.7)
Invalid or inconclusive	1 (4.5)
Positive result expectation (*n* = 16)	
Expected, wanted a negative result	11 (68.8)
Unexpected, wanted a negative result	3 (18.8)
Expected, wanted a positive result	2 (12.5)

Among participants who previously used FTS (*n* = 31), the average amount of time using FTS was 1.7 years (range 1 month to 5 years). When asked about the frequency of FTS use, answers from participants who had used FTS in the past varied. Ten participants reported using FTS one to four times a month, and two participants reported using FTS daily. Six participants reported only ever using FTS a few times. Three participants reported that they no longer used FTS. One of them said:

At first, in the beginning it was more I used them a lot. But as time went by I slowed down […] Because I basically knew the results of everything now.

Stephanie, female, Black, crack cocaine

One participant described previously using FTS on heroin/fentanyl and recently starting using FTS again because of suspected fentanyl adulteration of methamphetamine,

Like, I would use them a couple times here and there but … recently I've used them, like, two or three times maybe, just because I want the meth. Why am I falling asleep, you know what I mean?

Derrick, male, White, meth

When asked about the most recent FTS result of a non‐heroin/fentanyl sample (*n* = 22), 73% reported it was positive; among these, a majority (69%) expected it to be positive but wanted it to be negative. Out of the participants who had previously used FTS, two‐thirds had only used them on heroin/fentanyl.

Most participants knew at least one other person who used FTS and nearly two‐thirds said they had been present when another person was using FTS on a drug. Among participants who had used FTS with others (*n* = 26), nearly half said that they were usually the one testing the drug sample.

One participant reported that if he was the one using the test strip, it was usually because he was teaching someone else how to use it,

If they don't know what they're doing, I'll offer to do it.

Thomas, male, White, Rx pill

Another participant stated that she helped teach someone who was using the strip incorrectly.

I think I did actually show someone before, now that I'm thinking about it … sometimes, people will try to [use FTS], and then not add water to it.

Sarah, female, White, powder cocaine

Most participants had conversations about FTS with at least one other person (65%). Discussions on FTS within personal networks of PWUD included recommending FTS to another person. One participant said,

Yeah. I bring it up to people all the time. I'm like, “Yo, you're not doing what you think you're doing.” I get ‘em as often as I can. And I'll hand ‘'em to people, like, “Check it out, check it out, so you know what you're doing.

Michelle, female, White, crack cocaine

Participants noted that a significant barrier to FTS usage was a lack of general understanding surrounding how to use them correctly. Three participants reported general difficulties in comprehending how to use the strips because of a lack of instruction or a lack of detail, while another three participants specifically reported that they felt unsure of how to correctly read or interpret the results. One participant who thought you had to peel part of the FTS to use it said,

At first I didn't know how to use it. So I just gave up on it, but I didn't know that you had to break it and stick the soft part into the water. I didn't know that. I'm like, “Why do they give you this thing and don't say how to use it?”.

Aaron, male, Black, methamphetamine

There were several barriers that related to practical concerns, including one participant expressing PWUD's reluctance to ‘waste’ substances by using them to test. The ‘flimsy’ nature of the test stick was also considered a deterrent by one participant. The branded packaging was specifically cited as confusing by two participants. Conversely, several factors were identified that promoted FTS usage among the population. Aspects like user‐friendly and simplistic design as well as ease of use were cited by 13 participants, with five participants expressing that their familiarity with pregnancy tests assisted them in utilisation and interpretation.

Easy access to FTS was mentioned by three participants, and five participants specifically cited local organisations as a valuable resource both for obtaining FTS as well as providing FTS education. FTS education through resources like booklets was cited by one participant as a reason she began using FTS.

### 3.2 FTS Simulations

During the drug checking simulations (Table 3), none of the participants in the study completed all the steps of using FTS according to best practice recommendations. Out of 13 items on the checklist, the median number of errors was 10.5 (range 0.7–13). Fourteen participants (43%) made some kind of error when placing the FTS in the drug, which included over‐submerging the FTS in water, putting the wrong end into the solution, and not submerging it into a solution at all (e.g., putting drops of solution onto the FTS). Only seven participants (18%) held the FTS in the solution for 15 s while 10 participants (25%) waited at least 3 min before interpreting the FTS result. Half of the participants were able to correctly interpret the FTS results. At least four participants noted from their test result, and the research team confirmed, that some of the results on simulated drugs—none of which contained fentanyl—were positive.

**TABLE 3:** Results of fentanyl test strip simulation.

Drugs prepared to be snorted or smoked										
DoC^a^	Pseudonym	1a^b^	1b^c^	1c^d^	1d^e^	2a^f^	2b^g^	2c^h^	3^i^	Notes
Cocaine	Stephanie	No	Yes	Yes	Yes	No	No	No	No	Initially forgot to add drug then added more than 10 mg when prompted by facilitator. Submerged past blue line for > 15 s. Read when water was rising (< 30 s).
Cocaine	KO	No	Yes	No	No	Yes	No	No	Yes	> 10 mg, 3 drops of water, didn't stir. Peeled part of the strip off, put on table and read after < 1 min.
Cocaine	Sarah	No	Yes	No	No	No	N/A	No	Yes	6 drops water into cooker, dripped water onto strip, put strip in bag of drugs and added more water. Tapped strip on table, laid on table for 1 min.
Cocaine	Maria	No	Yes	No	No	Yes	No	No	Yes	Used ½ tsp., > 10 mg drug, didn't stir. Left strip in cooker & read like that.
Cocaine	Nelson	No	Yes	No	No	Yes	No	No	Yes	20 mg drug, 2 ml water, put strip tip in, left it in cooker, read at 45 s.
Cocaine	Kiki	Yes	Yes	No	No	Yes	No	Yes	No	Added 5 drops water, did not mix, left strip in solution until line appeared then read.
Cocaine	Jason	N/A	N/A	N/A	N/A	N/A	N/A	N/A	N/A	Put test strip under tongue.
Cocaine	T	No	Yes	No	No	No	No	No	No	40‐50 mg drug, 3 drops water, bent wrong end of strip into water for 1.5 min. Peeled paper strip back.
Cocaine	Emma	No	Yes	No	No	Yes	Yes	No	No	10 drops of water, held strip after dipping for 15 s, peeled off white end & then read.
Cocaine	Syy	No	Yes	No	No	Yes	No	No	Yes	Put 30 cc water in first, added 20 mg drug, shook to dissolve. Submerged for 8 s, read results after 1.5 min.
Cocaine	Eric	No	Yes	No	Yes	No	No	No	Yes	Put in 10 mg drug, used 2 ml water & stirred with finger, then added more drug. Put wrong end of strip in, stirred and waited for water to go up (it didn't because wrong end).
Crack	Jeffrey	No	Yes	No	Yes	No	No	No	No	> 10 mg, ~10 drops water, put blue end in cooker, peeled off blue part, held in hand until reading.
Crack	Tiffany	No	Yes	No	No	Yes	No	No	No	Wicked up all water, held in hands to read, then rested in cooker diagonal.
Crack	Jade	No	Yes	No	Yes	Yes	No	Yes	No	About a grain of rice amt of drug, crushed with syringe, filled ¼ cooker w/water, mixed w/back of syringe. Swirled strip around in cooker, pulled test strip apart, held in hand for result and waited 30 min before reading.
Crack	Alberto	Yes	Yes	Yes	Yes	No	No	Yes	No	Held in water for 30 s, then over‐submerged, then ripped blue end off. Left strip submerged while waiting for result.
Crack	Tony	Yes	Yes	No	Yes	Yes	No	No	Yes	Used ¼ tsp. water, held in liquid for 45 s, held in hand to wait for results.
Crack	Lyric	Yes	Yes	No	No	Yes	No	Yes	No	Crushed crack in cooker but chunks remained. Touched test end, water did not travel up strip (not enough).
Crack	Michelle	Yes	Yes	No	Yes	Yes	Yes	No	Yes	Added ½ tsp., touched test strip, read results in 30 s.
Crack	David 2	Yes	Yes	No	Yes	Yes	Yes	No	No	Used ¼ tsp., after submerging for 15 s shook strip until result.
K2/Spice	Alyssa	No	No	No	N/A	N/A	N/A	N/A	N/A	Peeled strip apart. No water added‐ could not use.
Meth	Dee	No	Yes	No	Yes	Yes	No	Yes	No	Used ~2 ml water. Small amt of drug but > 10 mg. Submerged ~5 s.
Meth	Aaron	No	Yes	No	No	No	N/A	No	Yes	> 10 mg, not enough water, peeled off blue part, wet the whole strip, held in hand until reading.
Meth	Jenna	No	Yes	No	Yes	Yes	No	No	Yes	¼ tsp. used, held in water for 1 min, then in hand & read.
Meth	James	No	Yes	No	No	No	No	Yes	No	1–2 drops water, poured it onto test strip, then touched both ends of test. Read after ~3 min.
Meth	Laura	No	Yes	No	No	Yes	No	Yes	No	Did not stir, was unsure which end to submerge. Correct side submerged for > 15 s, then laid flat on table and read at time.
Meth	Derick	No	Yes	No	Yes	Yes	No	Yes	Yes	> 10 mg drug, less than 1 tsp., dipped for less than 15 s., set down and read at 3 min.
Meth	Andrew Birch	No	Yes	No	No	No	No	No	Yes	Added ¼ tsp. water to cooker, then dropped a rock in and did not mix. Put wrong end in, then flipped it. Submerged for 3 s, then held until resulted.
Meth	Lacey	Yes	Yes	No	Yes	No	Yes	Yes	No	Used ½ tsp. water, bent strip inside the cooker, then followed instructions.

Drugs prepared for intravenous use								
DoC^a^	Pseudonym	1a^j^	1b^k^	2a^f^	2b^g^	2c^h^	3^i^	Notes
Cocaine	Jeff	No	No	No	No	No	No	Mixed 50 mg drug w/50 cc water, mixed w/plunger, tore blue part off. Started over, held in for 10 s, then dipped for another 25 s. Dipped the torn one. Read the first one after 30 s.
Cocaine	Tyler Durden	Yes	No	No	No	No	No	Prepped shot, added ~1/4 tsp. Pushed strip into cooker and left it in until read.
Cocaine	Tom	Yes	No	No	No	No	Yes	Prepped shot, dripped 4 drops of solution onto test strip, left on cooker for 1.5 min, read, waited and read again at 2.5 min.
Cocaine	Janet	Yes	No	Yes	No	No	Yes	Only added a few drops of water, drew up shot, did not add more water. Put strip in cooker and waited for results (not enough water).
Crack	Nikki	Yes	No	No	N/A	No	No	Used ~50 mg drug and ~4 mL water. Squirted 1 ml of mixture onto test strip, which was flat on ground. Waited ~1.5 min.
Crack	Michelle 2	Yes	No	Yes	Yes	Yes	Yes	Drew up shot and left some solution in cooker. Did not add water.
Meth	Pinky	No	No	Yes	No	No	Yes	Used 5 drops of water, > 10 mg drug, drew up most liquid in syringe. Stirred strip in cooker in remaining drop of water. Waited 5 mi, added more water when no lines showed up.
Rx pills	Reed	Yes	Yes	Yes	No	No	Yes	Prepped shot, added correct water. Handled both ends of strip, held in water for 2.5 min, held in hand to read.

Drugs in pill form									
DoC^a^	Pseudonym	1a^l^	1b^d^	1c^e^	2a^f^	2b^g^	2c^h^	3^i^	Notes
Rx pills	David 1	No	No	Yes	No	No	No	No	Broke pill in ½. Poured desiccant over it. Mixed ½ pill with full cooker of water. Stirred handle in water for 30 s. Shook for 30 s. Left on ground briefly, then picked up and shook again.
Rx pills	Thomas	Yes	No	No	Yes	Yes	No	Yes	Broke pill in ½, scraped 10 mg into cooker. Added 3 drops of water, swirled. Touched both end of strip, held in 15 s, laid flat on cooker, read at 2 min.
Rx pills	Mia	No	No	Yes	Yes	No	No	Yes	Put 30 mg and 3 drops water, mixed, then added more water. Touched both ends of test strip, inserted solution for 10 s & read at 30 s.
Rx pills	Scotty	Yes	No	Yes	Yes	Yes	No	Yes	Crushed ½ pill, used ½ tsp. water, held strip in 15 s, then held in hand to read.

^a^ Drug of choice, simulation substance selected.

^b^ Used correct amount of drug for the solution being tested.

^c^ Put substance into container/cooker.

^d^ Added correct amount of water to solution.

^e^ Stirred substance into water until dissolved.

^f^ Held the blue end of the strip, put it into the solution up to the water mark.

^g^ Allowed liquid to travel up the strip for 15 s.

^h^ Removed the strip and waited 3 min to interpret the result.

^i^ Read fentanyl test strip result correctly.

^j^ Prepared shot and set syringe aside.

^k^ Added 1 mL of water into container/cooker.

^l^ Used enough substance from crushed tablet or scraped from the middle of the pill.

#### 3.2.1 Drugs Prepared for Snorting or Smoking

Twenty‐eight participants (70%) chose to do a simulation with a drug that was being prepared to be snorted or smoked. Substances included powder cocaine (39%), crack cocaine (29%), methamphetamine (29%) and synthetic cannabinoids (4%). Only 25% of participants used the correct amount of drug sample needed to complete a FTS assessment. Over half of participants (57%) used too much of the drug sample in the solution (> 10 mg). Additionally, most participants (93%) did not use enough water to dilute the sample (< 1 tsp). More than half of participants (54%) did not stir the substance into the water before using the FTS. Twelve participants (43%) did not correctly put the FTS into the solution: five participants put the wrong end of the FTS into the water, four participants submerged the strip past the water mark and three participants did not submerge the strip at all. Once submerged, 10 participants (36%) pulled the FTS out of the solution in under 15 s and seven participants (25%) left the FTS in solution for more than 15 s. Half of participants read the FTS results in under 3 min after pulling it out of the solution and over half (54%) did not correctly interpret the results of the FTS.

#### 3.2.2 Drugs Prepared for Intravenous Use

Eight participants chose to do a simulation with a drug that was being prepared for intravenous use. Substances included powder cocaine (*n* = 4), crack cocaine (*n* = 2), methamphetamine (*n* = 1) and prescription pills (*n* = 1). Over half of the participants (57%) used too much of the drug sample in the solution (> 10 mg). Only one participant put the correct amount of water into the cooker after preparing a syringe for injection; nearly a quarter added less than 1 mL of water into the cooker to submerge the FTS. Four participants (50%) did not correctly put the FTS into the solution. Only one participant submerged the FTS in the solution for 15 s. Most participants (75%) waited at least 3 min before reading the results of the FTS. Three participants (38%) did not correctly interpret the results of the FTS.

#### 3.2.3 Drugs in Pill Form

Four participants (10%) chose to do a simulation with an imitation drug that was in pill form for an oral route of administration. Two participants (50%) put the correct amount of drug into the cooker to test. None of the participants used enough water to dilute the sample; all of them used less than 12 mL. One participant (25%) put the wrong end of the FTS into the solution for testing, two participants held the FTS in the solution for 15 s and all of the participants interpreted the results of the FTS in under 3 min. Three out of four interpreted the result correctly.

### 3.3 Interpretation of FTS Result Photographs

When asked to interpret the results in the first image of a faint negative FTS (Figure 2), most participants (73%) incorrectly interpreted it as a positive or invalid result. The second FTS image showing a positive result (Figure 3) was incorrectly interpreted as a negative or invalid result by 55% of participants. The third strip of a negative FTS result (Figure 4) was incorrectly interpreted as a positive or invalid result by 45% of participants.

## 4 Discussion

In this multi‐step study about the use of FTS, all participants simulating FTS used them incorrectly in at least one way. While FTS are increasingly promoted as an important tool in preventing overdoses [13], promotion is rarely accompanied by statements that training is essential to using FTS correctly.

FTS are a relatively straightforward tool, one that PWUD believe is easy and beneficial to use [14, 15, 16]. Participants in this study echoed findings from other studies about ease of use, with some saying that they train others to use FTS. This means incorrect practices are being disseminated to others; proper training would ensure that people can train others in proper use.

Half of the sample was never trained to use FTS; among these, many relied on the back of the FTS package, which only included images of positive and negative results. The company distributing the FTS used in this study recently updated packaging to show results more clearly and has since provided a QR code to a demonstration video, though this requires access to technology. More commercial brands of FTS are emerging and may present packaging differently. Companies selling FTS have an ethical duty to provide clear, easy instructions for use directly on their packaging. These should be complete instructions that do not require internet access. Manufacturers should also recognise that FTS practices are in various environments, many suboptimal and unlikely to be in accordance with ideal laboratory practices.

Pictures of FTS results were incorrectly interpreted by over half the sample in photographs showing a faint negative and a positive line. Participants related an ease with reading test strips results due to familiarity with similar tests (e.g., pregnancy). However, the results are often the opposite of what is shown in pregnancy and rapid COVID‐19 tests, in which two lines indicate a positive result. This may be leading some to misinterpret results. Training should emphasise how results differ from other common at‐home tests and refer people back to instructions showing what the presence or absence of lines indicates. Training should also stress that the second line in a negative may be extremely faint and encourage people to read results in well‐lit conditions or to use the flashlight feature on a phone when available.

In failing to instruct PWUD on proper FTS use, potential risks for adverse outcomes follow. Many people who receive positive results are likely unable to dispose of a substance that cost them money and which they may need to alleviate withdrawal. Using a substance they believe contains fentanyl when they do not want to use fentanyl may be emotionally taxing or cause people to feel shame. People may no longer believe in the utility or accuracy of FTS. Additionally, continued drug use despite positive results may give people, especially those who do not regularly use opioids, false confidence about their tolerance levels. Future research should explore these potential ramifications. Second, continuously receiving a positive FTS result and proceeding to use a substance without experiencing harm may lead to alarm fatigue. Alarm fatigue, a term often used in the medical field, refers to when a recurring alert leads to desensitisation to the alert itself [17]. Applied to the situation of multiple FTS false positives, alarm fatigue may lead someone to disregard a true positive FTS result, placing them at risk of an overdose. As indicated by one participant, repeated positives, which may be false, can lead to stopping FTS use altogether. Programs should consider not distributing FTS until staff can take time to demonstrate how to correctly use them. Verbal training should address common misconceptions and myths and be accompanied by written instructions, ideally with a QR code linking to video training.

PWUD in this study were applying the tools and information available to them to use FTS. They were curious to know about the composition of their drugs and highly motivated to take action to stay safer. Many also expressed a desire to train others due to motivations to help protect others from harm. PWUD operate as safely as possible given structural vulnerabilities and barriers to safer drug use and available resources. With FTS, they face a number of obstacles including a lack of training, variability in the quality of FTS and difficulty accessing enough strips to use consistently. FTS may be an empowering tool if paired with effective education. Further research should be conducted to better understand ideal training components and modes of delivery. Given that 20% of the sample was trained by another PWUD, future studies can assess diffusion of an educational intervention among PWUD with large social networks. This may be particularly effective as most PWUD in this study had tested with other people and had conversations with other PWUD about FTS.

A number of participants received a positive FTS result during the simulation; none of the simulation materials provided contained fentanyl. It is possible that they touched the testing end of the strip with hands that had recently prepared fentanyl for use prior to study .enrollment. Another potential explanation is that they were using so little water that there was a cross‐reaction with the strips, causing a false positive. Previous research has found that high concentrations of lidocaine, diphenhydramine, methamphetamine and MDMA can cause a false positive, but no study to our knowledge has yet assessed non‐psychoactive cutting agents that may cause false positives [18, 19].

A core issue with the use of fentanyl test strips is that it requires correct use in a multi‐step process, ideally with one FTS used each time a person uses a substance. This places the burden onto PWUD, many of whom may not be positioned to use FTS in ideal conditions every time they are preparing drugs for use. For example, withdrawal and outdoor conditions are barriers to FTS use [10, 16]. A safe and regulated supply of drugs would remove the onus from PWUD, ensuring that substances purchased without fentanyl are indeed free of fentanyl and other adulterants. Failing that, more comprehensive point of care drug checking, in which a person can submit a sample of drug for checking to a technician with spectroscopy or other more advanced drug checking technology, transfers the onus from PWUD to people with formal training in testing drugs and delivering results. The implications of self‐testing drugs are particularly salient as new test strips are developed for other substances, such as xylazine, although the use of multiple test strips may also require PWUD to prioritise one type of test strip over others due to time and variability in correct use.

This study is subject to several limitations. First, participants were recruited from the Kensington neighbourhood of Philadelphia and participants may differ meaningfully from other PWUD, as the majority were unhoused and reported regular use of multiple substances. Additionally, 40 participants using FTS on a variety of simulated drugs limited our ability to determine whether errors occurred by drug type selected. The sample was English‐speaking and predominantly unhoused, White, and comprised of people using multiple substances on a daily or near daily basis. While the study team attempted to replicate true testing conditions, participants were still demonstrating practices in a simulated environment and may have acted differently accordingly.

## 5 Conclusion

While FTS are increasingly distributed to PWUD to decrease overdose risk, all participants in this study incorrectly used FTS on simulated drugs. Training is essential to ensure this harm reduction tool is correctly and effectively used. Proper training should include sample preparation, mechanics of testing, and correct interpretation of results. Systematic point of care drug checking and a regulated, safer supply of drugs would obviate the need for FTS altogether.

## Author Contributions

**Megan K. Reed:** funding acquisition, conceptualisation, methodology, investigation, formal analysis, writing – original draft. **Tracy Esteves Camacho:** methodology, investigation, formal analysis, writing – original draft, project administration. **Kristin L. Rising:** supervision, writing‐review and editing. **Rose Laurano:** investigation, formal analysis, writing – review and editing. **Danielle Albaciete:** investigation, formal analysis, writing‐review and editing. **Stephen E. Lankenau:** supervision, writing‐review and editing.

## Conflicts of Interest

The authors declare no conflicts of interest.

## Data Availability

The data that support the findings of this study are available on request from the corresponding author. The data are not publicly available due to privacy or ethical restrictions.
